# The Iris Thickness in a Healthy Saudi Population

**DOI:** 10.7759/cureus.12521

**Published:** 2021-01-06

**Authors:** Yasir H Ziaul, Alka Mahale, Sejo Varghese, Farheen Khanam, Muneera AlFutaise, Muhammad A Ahad, Deepak P Edward, Rajiv B Khandekar

**Affiliations:** 1 Research, King Khaled Eye Specialist Hospital, Riyadh, SAU; 2 Anterior Segment, King Khaled Eye Specialist Hospital, Riyadh, SAU; 3 Ophthalmology and Visual Sciences, University of Illinois at Chicago, Chicago, USA; 4 Epidemiology and Public Health, King Khalid Eye Specialist Hospital, Riyadh, SAU

**Keywords:** iris, scleral spur, pupil, anterior segment optical coherence tomography (asoct)

## Abstract

Purpose

Iris thickness (IT), a known risk factor for angle closure glaucoma, has not been evaluated in the normal Saudi population.

Methods

Quantitative information on IT was evaluated in healthy Saudi eyes using anterior segment optical coherence tomography (ASOCT). IT and iris volume was measured with the room ‘light on’ (LON) and ‘light off’ (LOFF) using Image J software. IT in the nasal and temporal iris was measured at 500 µm (IT500) and 750 µm (IT750) from the scleral spur (SS). Differences in IT measurements by age, gender and type of refractive error were evaluated.

Results

We included 100 eyes of 50 healthy adult Saudis without ocular disease other than refractive errors. The mean age of 56 males and 44 females in the study group was 41.7 ± 14.5 years. The refractive status was as follows: emmetropia (35 eyes), mild/moderate myopia (33 eyes), high myopia (17 eyes) and hyperopia (15 eyes). The IT750 with LON was significantly more than IT500 both nasally (P = 0.03) and temporally (P < 0.001). The difference in IT750 and IT500 with LOFF was significantly more nasally (P = 0.03), temporally (P = 0.02), and with LON nasally (P = 0.005). IT was thicker in males when compared to females and variation of IT by refractive error was significant but not by age. The mean pupil diameter and anterior chamber depth decreased with age (P < 0.001). Anterior chamber width was not affected by age or illumination.

Conclusion

The baseline iris thickness in the Saudi eyes could be used to compare iris thickness in eyes with angle closure glaucoma among the Arab population.

## Introduction

Ocular biometry is essential for understanding ocular growth and pathogenesis. Changes in anatomic structures may cause visual abnormalities and its modulation influences both onset and progression of disease [1]. Anterior segment optical coherence tomography (ASOCT) is increasingly being used for scanning and imaging the cornea, anterior chamber, chamber angle, iris, lens and anterior vitreous [2]. It can provide quantitative data from anterior segment structures including the cornea, iris and anterior chamber [3].

Iris morphology and the anterior chamber angle anatomy play an important role in the pathogenesis of various forms of glaucoma [1]. Peripheral iris thickness and curvature are vital parameters that have helped us in understanding the pathogenesis of different types of glaucoma (especially angle closure) [4]. Nongpiur et al. used ASOCT to assess patients with angle closure glaucoma and reported smaller anterior chamber width (ACW) [4], anterior chamber area (ACA), and anterior chamber volume (ACV) as well as increased iris thickness, area, and curvature. These researchers and others also found that changes in iris volume during dilation and larger lens vaults were associated with primary angle closure glaucoma [4,5]. Huang et al. found that iris thickness and area in normal eyes were significantly associated with age, anterior chamber width [1], and pupil diameter. Lin et al. measured anterior segment parameters including iris thickness (the perpendicular distance from iris pigment epithelium to the anterior iris surface) at 500, 750, and 1000 μm from the iris root [6], and found that iris curvature in low illumination influenced the changes in iris area and pupil diameter, which may contribute to disease progression. It has been suggested that orbital and ocular parameters could vary with ethnicity [7]. Therefore, ASOCT measurements of normal anterior segment structures in different populations would be useful in understanding the pathophysiology of diseases such as glaucoma and help us in improving clinical detection of disease or identifying risk factors [1, 8, 9].

In this study, we present data on iris thickness and iris volume in healthy Saudi individuals without ocular disease. We also present variation in iris volume in relation to illumination and other factors like refractive error, gender and age.

## Materials and methods

The institutional research and ethics board approved this cross-sectional study (1365-P). All participants received a detailed explanation about the study and signed an informed consent form. This study adhered to the principles of the Declaration of Helsinki. Individuals were included in the study if they were between 15 and 80 years old, presenting for routine screening of refractive error and had no major eye disease. A person with a history of previous eye surgery, ocular trauma, history of topical/systemic medications or conditions that could interfere with iris anatomy or physiology, glaucoma, laser treatment and a cup-to-disc ratio of more than 0.4, intraocular pressure (IOP) ≥ 22 mmHg, peripheral anterior synechia (PAS) or any factor limiting normal ASOCT imaging of the anterior segment was excluded.

Demographic data including age and gender were collected on a pretested data sheet. All participants underwent a standardized eye examination that included visual acuity measurement, slit-lamp bio-microscopy, intraocular pressure (IOP) measurement by Goldmann applanation tonometry (Haag-Streit, Köniz, Switzerland) and posterior segment examination with a 78-diopter lens (Volk Optical, Mentor, OH, USA) (undilated pupil). The refractive error was assessed using cycloplegic refraction when indicated followed by subjective correction. Emmetropia was defined as refractive error between <−0.5 D to <+0.5 D, myopia was defined as refractive error ≥−0.5 D and hyperopia as refractive error ≥+0.5 D. Myopia was further graded as low myopia (≥−0.5 to <−3D), moderate myopia (−3 D to −6 D) and high myopia (≥6 D) [10]. Hyperopia was further graded as: low hyperopia (≥+0.5 D to +2 D), moderate hyperopia (+2.25 D to +5 D) and high hyperopia (>+5 D). The spherical equivalent was calculated using formula spherical RE + half of the cylindrical RE [11].

Cornea/Anterior segment OCT (3D CAS-OCT SS-1000, Tomey Corp., Tokyo, Japan) was used for morphometric measurements [12]. The right eye (OD) and left eye (OS) were scanned, first with the room light on (LON) and then with the room light off (LOFF) (< 5 lux) for two minutes. All ASOCT images were acquired by an ophthalmic technician specifically trained to use this equipment. The participants were asked to fixate on an internal target in the machine and to keep the eye open for the duration of the scan (≈2.4 seconds). For each eye, the machine obtained a three-dimensional scan of anterior segment with eight radial slices (0°-180°, 23°-203°, 45°-225°, 68°-248°, 90°-270°, 113°-293°, 135°-315°, and 158°-338°). The scan quality was checked manually by the authors (FK, ZY, AM). A successful scan was defined as one that included the complete cornea, angle, iris and lens without artifacts. If motion artifacts or image artifacts were present due to the partial or complete eyelid closure or squeezing the eye, the image was discarded. The scan was repeated if more than four of the eight radial slices (including the horizontal 0°-180° meridian) were not clear. For this study, we used the radial scans at the 0°-180° axis (horizontal meridian) for the measurements. We selected this meridian as lid distortions could be avoided in the horizontal meridian and there is a higher chance of lid shadow in other segments [12].

Four scans from each individual (OD/OS/LON/LOFF) were selected for analysis. The scleral spurs (SS) on the nasal and temporal aspects of the scan were marked in each eye by the ophthalmologist (ZY). The scleral spur anatomically represents the junction between the inner wall of the trabecular meshwork and the sclera and is marked by a prominent inner extension (inward protrusion) of the sclera with a change in curvature of its inner surface [13]. This was identified in this study based on Nakakura et al. [14].

**Figure 1:**
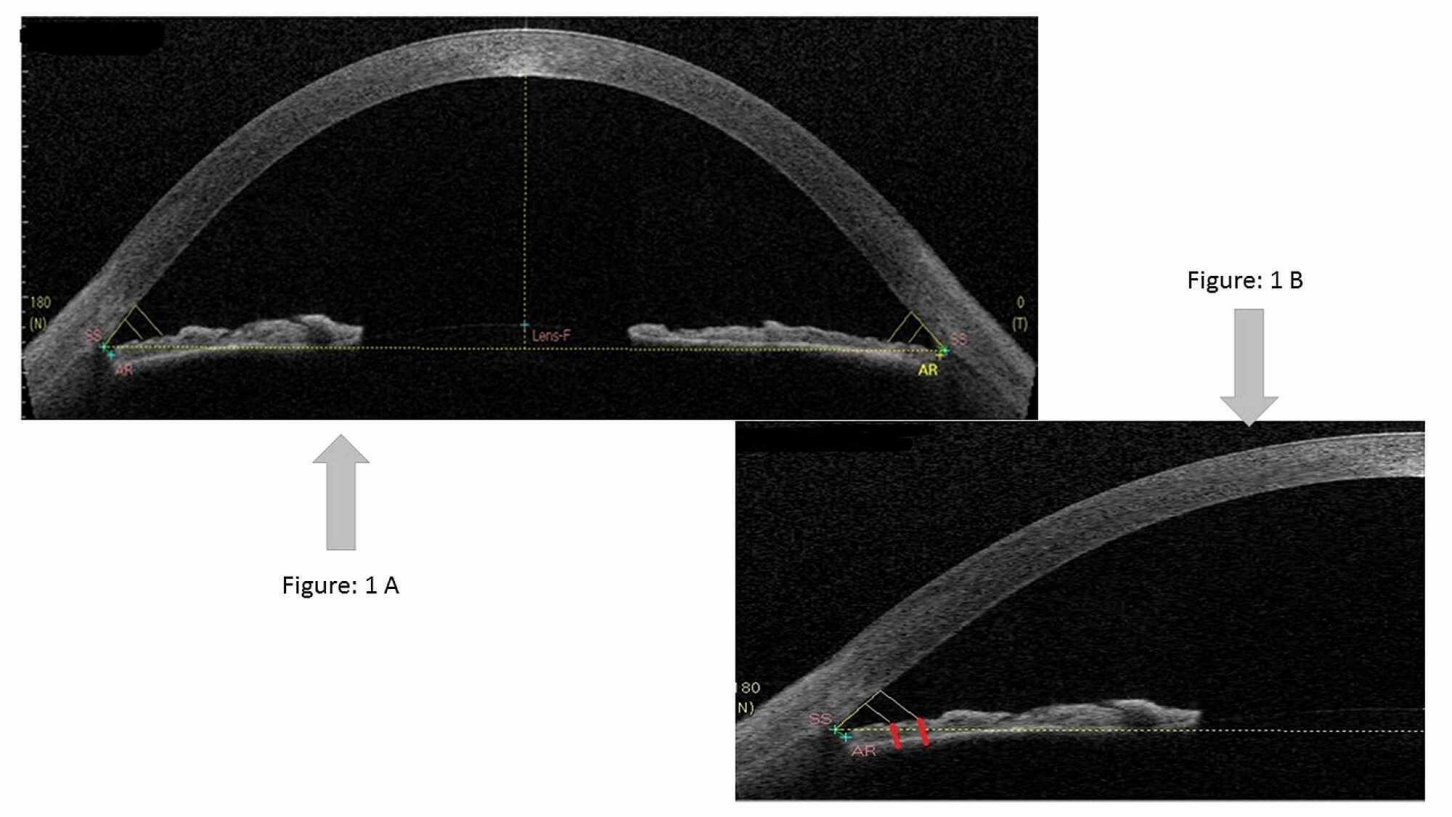
Iris thickness measurement at IT500 and IT750 from the scleral spur on the nasal and the temporal side captured by anterior segment optical coherence tomography (ASOCT) and evaluated images of normal Saudi population using ImageJ software. 1A shows scan at 180° axis. 1B shows angle of anterior chamber on nasal side of 180° axis: IT500 and IT750 is represented by two red vertical lines shown in the figure. SS = scleral spur; AR = Angle recess; Lens F = Front of lens

Iris thickness (IT) was measured at 500 µm (IT500) and 750 µm (IT750) from the scleral spur (Figure 1). These landmarks were defined as points intersected on the anterior surface of iris by lines perpendicular to the plane of the trabecular meshwork at the indicated distances (500 µm and 750 µm) anterior to the SS (add references). The Casia software automatically marks these points once the SS and angle recess has been defined by the observer. The software also marks and provides a measure of anterior chamber width (ACW) from the nasal end of SS to the temporal end of SS. The anterior chamber depth (ACD) was measured as distance from the posterior corneal surface to the anterior lens capsule (along the anatomical/optical axis) [15,16].

The image was saved and transferred to ImageJ 1.51k, a Java-based image processing program developed at the National Institutes of Health and the Laboratory for Optical and Computational Instrumentation (LOCI, University of Wisconsin, USA). We used this software for measurement of the iris thickness and volume [17]. A reference image obtained with the Casia machine was calibrated for the pixel size to define a distance of 1-mm scale bar (in this case 81.5 pixels = 1 mm) (Figure 1). This image was saved and used to calibrate the ImageJ software prior to performing iris thickness measurements to minimize measurement bias as follows: The scale was set as distance of pixel 81.5, known distance 1, pixel-to-aspect ratio 1. Measurements were performed in millimeters. The ACW and ACD were measured again with ImageJ and compared with the Casia readings to verify calibration accuracy. Iris thickness was calculated as the perpendicular distance from iris pigment epithelium to the anterior iris surface (both appear as hyper-reflective layers on OCT) as outlined above using the markings obtained from Casia. Subsequently, measurements were performed on the four scans from the study subjects (OD/OS/LON/LOFF).

Iris volume was measured between IT500 and IT750 from the SS. This particular area is trapezoidal in shape. The upper and lower surfaces of the iris represent sides of trapezoid. The iris thickness at 500 and 750 µm and IT750 are width of trapezoid. The height (H) of the trapezoid is the distance between IT500 and IT750 (250 µm) and the length of the trapezoid (L) was the circumference of a circle with radius from the center of pupil to the center point between IT500 and IT750 (625 µm away from SS). The formula to calculate iris volume was (Volume of the Trapezoid) = L x H x (IT500+IT750/2) [18, 19].

The data were collected on a pretested collection form and subsequently entered into an Excel® spreadsheet (Microsoft Corp., Redmond, WA, USA). The data following review was transferred to the Statistical Package for Social Studies (SPSS v.22; IBM Corp., Armonk, NY, USA). The frequencies and the percentage proportions were calculated for qualitative data. For quantitative data, normality was tested, and the mean and standard deviations were calculated if the variable was distributed normally. To study the associations between the various parameters measured, matched pair analysis was performed and the Odds Ratio (OR) was estimated. The 95% confidence interval (CI) was calculated and a two-sided ‘P’ value was calculated. A P-value <0.05 was considered statistically significant.

## Results

The study sample comprised 100 eyes of 50 healthy Saudi individuals. There were 28 males and 22 females. The age of 26 subjects ranged between 17 and 40 years and the remaining 24 were above 40 years in age. The mean age was 41.7 ± 14.5 years. The refractive status of the eyes was as follows: emmetropia (n = 35), mild/ moderate myopia (n = 33), highly myopia (n = 17) and hyperopia (n = 15).

**Figure 2:**
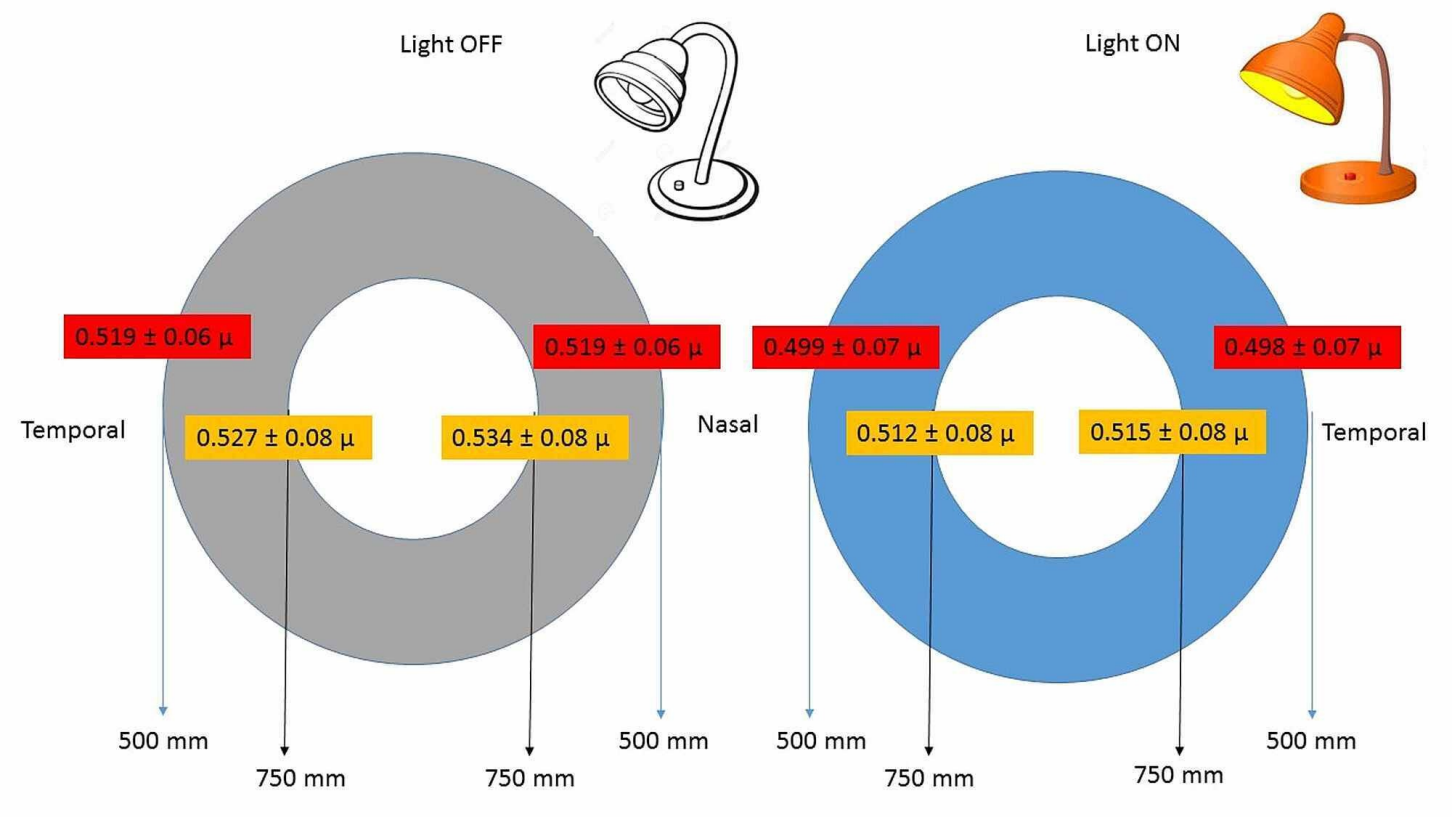
Iris thickness measured by anterior segment optical coherence tomography (ASOCT) using ImageJ of 100 Saudi healthy eyes. Grey shaded figure is in light OFF and blue colored picture is in light ON conditions The value is mean and two ends of the vertical bar are upper and lower limits of 95% confidence interval. Locations are on nasal and temporal side of 180° axis of the scan at IT500 and IT750 distance from scleral spur.

The iris thickness at IT500 and IT750 nasal and temporally in the light ON and light OFF are shown in Figure 2. The IT750 with LON was significantly greater than IT500 both nasally (P = 0.03) and temporally (P < 0.001).

Iris thickness parameters in healthy Saudi eyes by refractive error status are shown in Table 1.

**Table 1:** Iris thickness parameters measured using scleral spur as reference in healthy Saudi eyes by refractive error.

Temporal side of horizontal axis	Emmetropia (N = 35)		Mild-Moderate Myopia (N = 33)		Mild-Moderate Hyperopia (N = 15)		High myopia (N = 17)		Two-sided P-value
Light ON	Mean	SDV	Mean	SDV	Mean	SDV	Mean	SDV	
At 500 µ away	0.483	0.08	.495	0.08	0.546	0.07	0.473	0.07	0.02
At 750 µ away	0.496	0.08	.520	0.08	0.576	0.08	0.489	0.06	0.003
Light OFF									
At 500 µ away	0.485	0.07	0.534	0.07	0.557	0.08	0.484	0.06	0.004
At 750 µ away	0.504	0.06	0.543	0.08	0.586	0.06	0.489	0.06	0.001
Nasal side of horizontal axis									
Light ON									
At 500 µ away	0.488	0.08	0.510	0.06	0.518	0.06	0.482	0.05	0.3
At 750 µ away	0.487	0.09	0.525	0.08	0.566	0.06	0.484	0.05	0.005
Light OFF									
At 500 µ away	0.511	0.07	0.511	0.06	0.550	0.07	0.510	0.05	0.1
At 750 µ away	0.517	0.07	0.543	0.08	0.580	0.06	0.512	0.05	0.01

IT was thinnest in eyes with high myopia. The variation in iris thickness by different types of refractive error at IT750 was statistically significant (P = 0.001).

Iris thickness parameters in healthy eyes of Saudi males and females are shown in Table 2.

**Table 2:** Iris thickness parameters measured by using scleral spur as reference in healthy eyes of Saudi males and females.

Temporal side of horizontal axis	Male (N = 56)		Female (N = 44)		Two-sided P-value
Light ON	Mean	SDV	Mean	SDV	
At 500 µ away	0.499	0.08	0.489	0.08	0.5
At 750 µ away	0.525	0.08	0.501	0.07	0.4
Light OFF					
At 500 µ away	0.519	0.08	0.507	0.06	0.05
At 750 µ away	0.533	0.08	0.519	0.06	0.05
Nasal side of horizontal axis					
Light ON					
At 500 µ away	0.506	0.07	0.489	0.06	0.3
At 750 µ away	0.516	0.09	0.508	0.06	0.007
Light OFF					
At 500 µ away	0.526	0.06	0.59	0.07	0.3
At 750 µ away	0.538	0.08	0.529	0.06	0.05

The iris was significantly thinner in females compared to males in the lights OFF condition at the IT750 position.

The IT750 on the temporally was significantly thinner and correlated with the age of the Saudi participants both in LON (Pearson r = -0.2; P = 0.05) and LOFF (Pearson r = -0.4; P < 0.001) conditions.

**Figure 3:**
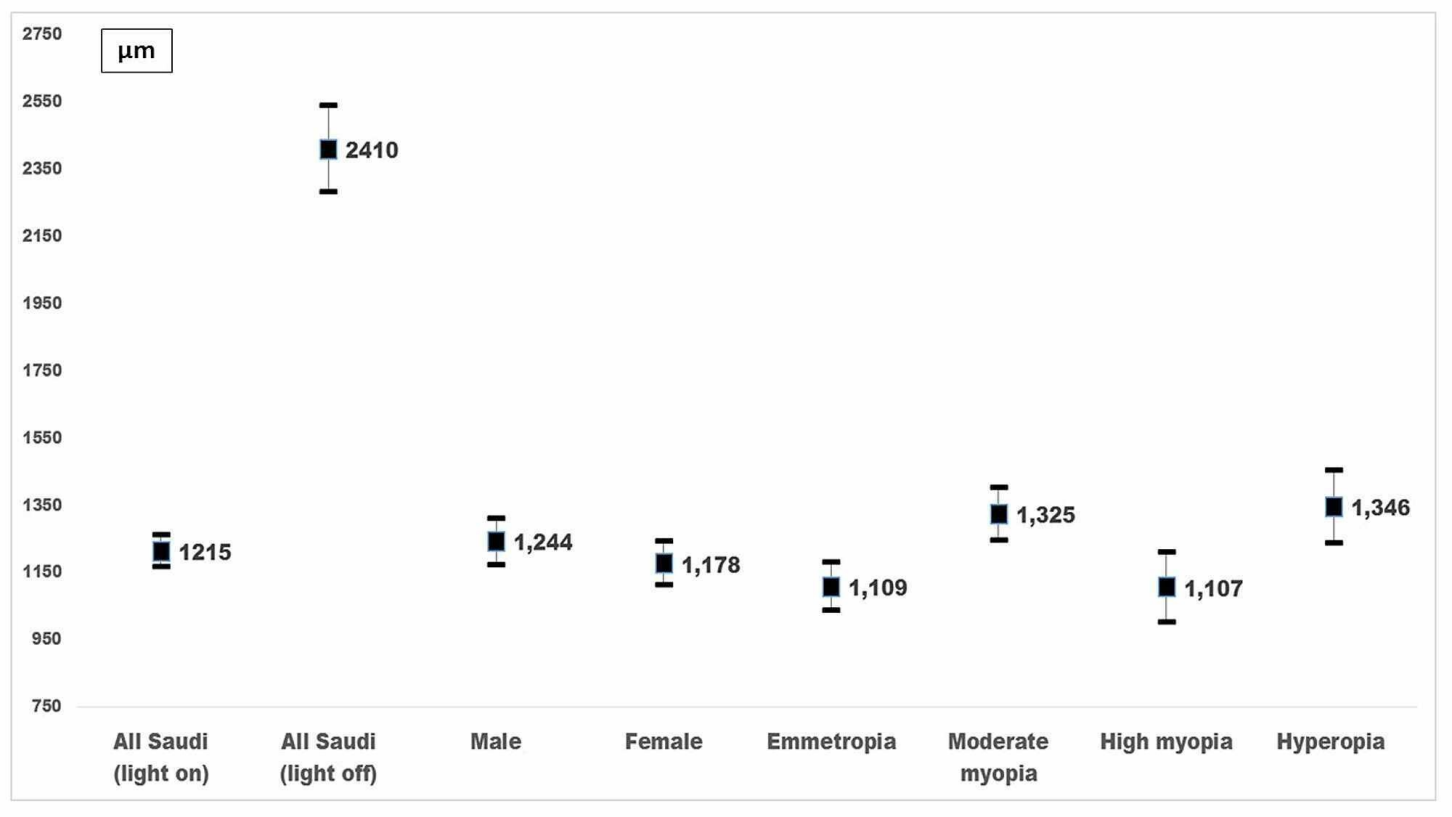
Iris volume (IV) measured by formula of volume calculation of a trapezoid using anterior segment optical coherence tomography (ASOCT) of 100 Saudi healthy eyes. The central value (µm^3^) is the mean and two ends of the vertical bar are upper and lower limits of 95% confidence interval.

The nasal and temporal iris volume under different lighting conditions is shown in Figure 3. The mean iris volume was 1215 ± 236 µm^3^ in LON and 2410 ± 647 µm^3^ in LOFF. The difference was significant (P < 0.001). Iris volume had a positive linear correlation with gender (P < 0.001) and RE with LON and LOFF (P < 0.001) suggesting that while reviewing iris thickness, age and refractive status should be taken into account. The linear correlation suggested that age was negatively correlated to iris volume in both LON (Pearson r = -0.51; P < 0.001) and LOFF conditions (Pearson r = -0.56; P < 0.001).

The mean pupil diameters with LON and LOFF were 5.0 ± 1.1 mm and 5.8 ± 1.2 mm, respectively. The mean difference in pupil diameter was 0.8 mm [(95% CI: 0.7; 0.9) P < 0.01]. Pupil diameter significantly decreased with age under LON condition (Pearson r = -0.53, P < 0.001) and with LOFF (Pearson r = -0.64, P < 0.001). The mean ACW was 12.1 ± 3.1 mm. The mean anterior chamber depth was 3.1 ± 0.4 mm which significantly decreased with age in both with LON and LOFF condition (Pearson r = -0.6, P < 0.001).

## Discussion

In this study, we evaluated ocular biometry of iris using Swept source OCT in an Arab population to understand iris morphology in a clinical setting. In the normal eyes enrolled in this study, iris thickness measured with SS as reference at 750 μm seems to provide a more consistent value. Both thickness and volume of the iris were lowest in high myopic and highest in the hyperopic eyes. Both thickness and the volume of the iris were more in males compared to females.

The data presented in this study could be used as a reference for future studies and will enable the evaluation of differences in iris morphology between ethnicity and its role as a potential risk factor for glaucoma. The data presented here provides a basis to compare different iris parameters of healthy population to the eyes with pathology such as angle closure glaucoma and with narrow angle including plateau iris.

IT among Saudis (Arabs) is thicker than that reported among African Americans, Caucasians (IT 750; 0.44 ± 0.1 mm) as well as Chinese population (IT-750; 0.46 ± 0.08 mm) [1, 7]. Hence the nomogram data for an instrument needs to be re-calibrated when such instrument is used for measuring parameters of Saudi (Arab) population. It is possible that the thickness of the iris may play a role in primary angle closure glaucoma (PACG), which is more common in Saudis by causing narrowing of the anterior chamber angle [20].

In our study, iris thickness was measured only along the horizontal axis nasally and temporally. Other studies have included iris measurements in superior and inferior quadrant also [21, 22]. The laser interventions are usually carried out in upper quadrant of iris in sitting position using slit-lamp bio-microscope. Therefore, information on IT in upper quadrant would be useful to clinicians in effective iridotomy.

We found that the iris is thicker at IT750 compared to IT500 in different settings. This observation concurs with Liu et al. findings that report better correlations at 750 µm than 500 µm in ACG cases [9]. Nongpiur et al. also used the 750 µm location in their study and so did Huang et al. while evaluating normal individuals [1,4]. Hence our observations and those of previous studies indicate that IT750 is a good reference point for studies that measure iris thickness.

At low illumination, iris thickness and volume in the defined area was significantly higher when compared to measurements at high illumination. Our findings matched with the results of Hirose et al. [22]. But in contrast, Quigley et al. noted decrease in the volume after dilation [5]. It should be noted that the later study was based on small sample and change in pupil was following change in illumination as well as pharmacologic dilation in eyes with light iris pigmentation (European) was reviewed. A peripheral iris shift when pupils dilate in low illumination explains increase in thickness and volume of iris in low illumination.

IT with LON and LOFF among various refractive status was more constant and significant at IT750 compared to IT500. Highly myopic eyes had the thinnest iris, emmetropic eyes and eyes with mild to moderate myopia had intermediate IT and hyperopic eyes had the thickest iris. This finding is not surprising as the globe is longer with tissue structures stretched (in myopia) or compacted (in hyperopia) depending on axial length. The iris thickness in myopic eyes but with open angle in Singaporean study was 0.7 ± 0.13 mm and was not significantly different from IT in myopic eyes of our study [23]. O’Donnell et al. also noted that the biometric parameters like anterior chamber depth [24], horizontal iris diameter in myopic were higher compared to non-myopic eyes. Based on this observation, it is possible that iris thickness and not just a shallow chamber from altered anatomy in hypermetropia plays a role in narrowing of the anterior chamber angle in hypermetropia and conversely widening of the chamber angle in myopia. Consistent with our findings in the iris, Nishi et al. evaluated the choroid, which is another uveal tissue [25], and found that it was thicker in the hyperopic eye compared to the normal fellow eye. Ikuno and Tano reported a thinner choroid in high myopic eye [26]. As most studies have reported on the choroidal thickness, further studies are warranted to evaluate our observations of iris thickness and refractive error.

In our study, iris was thicker in males than in females. Huang et al. reported female eyes had lower values of different anterior segment biometric parameters including iris thickness among healthy Chinese individuals [1]. In study of twins, in contrast to our study, He et al. found that iris thickness was thinner in females than in males in Chinese population [27]. Furthermore, Invernizzi et al. reported that gender did not influence iris thickness in an Italian population (Caucasians). It is likely that the differences in anterior biometric parameters supported by Invernizzi et al.’s hypothesis that gender variation in iris thickness could be related to sex-determined anatomical variations during growth of the face [28].

In our study, iris thickness was negatively correlated to age. Huang et al. also found that iris thickness was negatively correlated to age in Chinese individuals [1]. Invernizzi et al. (Caucasian study sample) and He et al. noted no differences in iris thickness based on age in Chinese population [27, 28]. It seems that age is a modifying factor for iris thickness but varies by ethnicity. Further studies are required to confirm this correlation.

The pupillary diameter was larger in younger subjects compared to older subjects in our study. It however showed no relationship with gender, refractive error or iris thickness. The smaller pupillary diameter is likely due to age-related miosis which has been previously reported [24].

In our study, anterior chamber width was not affected by age or illumination. Our observations concur with previous literature [26, 27]. It is possible that the globe size, especially the anterior segment, reaches to the maximum size by 13 years of age [29].

We found that anterior chamber depth decreased with age. This observation is similar to that reported in previous studies. The decrease in depth could be due to an increase in lens thickness with age [1, 19]. The ACD did not vary by illumination.

We measured iris volume based on iris thickness measured at IT500 and IT750. This measure should not be compared with total iris volume based on total iris width. The difference in iris volume by illumination in our study was 1195 µm^3^. Invernizzi et al. used iris width from iris root to the pupillary margin which could be more effective in studying the impact of different diseases of the anterior chamber on iris volume (IV). IV measurements are challenging to interpret because they can vary widely with ambient illumination [28]. In our study, iris volume did not change significantly with age. Invernizzi et al. also found iris volume was unaffected with age in a Caucasian sample [28].

Maintenance of iris morphology and volume and no change in IV with age as noted by Invernizzi et al. [28], could be due to structural changes at molecular level [24]. IV was significantly greater in males compared to females. IV was larger in hyperopes than emmetropes and moderate myopic eye, and lowest in high myopic eye. Thus comparison of iris parameters (IT & IV) must also account for gender, age and RE of the eye.

So iris thickness and volume comparison with the whole iris should be interpreted with caution. We did not study the effect of accommodation on IT and IV due to logistic reason, changes in iris curvature and color of iris in normal healthy Saudi eyes.

## Conclusions

Anterior OCT was a useful and an easy tool to study iris parameters in normal healthy eyes. IT measured using the SS method at 750 µ can be used for comparison to eyes with different eye ailments. Iris parameters are influenced by illumination, refractive status, location on iris, gender and age of Saudi adults.
